# Behavioral profiling of SLC38A10 knockout mice using the multivariate concentric square field^TM^ test

**DOI:** 10.3389/fnbeh.2022.987037

**Published:** 2022-12-22

**Authors:** Frida A. Lindberg, Erika Roman, Robert Fredriksson

**Affiliations:** ^1^Department of Pharmaceutical Biosciences, Molecular Neuropharmacology, Uppsala University, Uppsala, Sweden; ^2^Neuropharmacology and Addiction, Uppsala University, Uppsala, Sweden; ^3^Division of Anatomy and Physiology, Department of Anatomy, Physiology and Biochemistry, Swedish University of Agricultural Sciences, Uppsala, Sweden

**Keywords:** behavior, exploratory behavior, MCSF test, phenotyping, SLC38A10, SNAT10, amino acid transporters

## Abstract

**Introduction:**

SLC38A10 is a gene that encodes the SLC38A10 protein, also known as SNAT10. The SLC38 family is evolutionary old, and SLC38A10 is one of the oldest members of the family. It is ubiquitously expressed, and its substrates are glutamine, glutamate, alanine, aspartate, and serine. However, little is known about its biological importance.

**Methods:**

In the current study, an SLC38A10 knockout mouse was run in the multivariate concentric square field*^TM^* (MCSF) test. The MCSF test gives the mouse a choice of areas to explore; sheltered areas, elevated and illuminated areas, or open spaces, and a behavioral profile is obtained. The multivariate data obtained were analyzed (i) for each parameter, (ii) parameters grouped into functional categories, and (iii) with a principal component analysis.

**Results:**

In the trend analysis, knockout mice had a decreased exploratory behavior compared to controls but did not show a distinct grouping in the principal component analysis.

**Discussion:**

There was not a pronounced difference in the behavioral profile in SLC38A10 knockout mice compared to their wild-type controls, although subtle alterations in zones associated with exploratory behavior and risk assessment in female and male knockout mice, respectively, could be observed. These results imply that a loss of function of the SLC38A10 protein in mice does not drastically alter behavior in the MSCF test.

## 1 Introduction

The *SLC38A10* gene is one member of the Solute Carriers (SLCs), which is the biggest group of transporters in humans (Hediger et al., 2013). Transporters and associated proteins are estimated to constitute around 10% of all human genes (Hediger et al., 2013), yet the characterization and deorphanization of these transporters are lagging behind (César-Razquin et al., 2015). SLC38A10 is one of these transporters which function is not yet fully understood. This transporter is one member out of eleven in the SLC38 family, which is a family of sodium-coupled neutral amino acid transporters, or SNATs (SNAT1-11), that is involved in a range of physiological functions. The SNATs are considered especially important for the glutamate-glutamine cycle in the brain (SNAT1, 2, 3, 5 and possibly 6, 7, and 8; Chaudhry et al., 1999; Boulland et al., 2002; Cubelos et al., 2005; Blot et al., 2009; Hägglund et al., 2011; Bröer, 2014; Gandasi et al., 2021), the glucose-alanine cycle between liver and muscle, and for the urea cycle (SNAT2, 3, 4, and 5; Chaudhry et al., 1999; Hatanaka et al., 2001; Varoqui and Erickson, 2002; Baird et al., 2004; Bröer, 2014). Less is known about the latter SNATs in the family, and the aim with the current study was to examine the importance of one of these transporters, SLC38A10 (SNAT10), for the behavior profile in mice.

SLC38A10 have a bidirectional transport of L-glutamine, L-alanine, L-glutamate and D-aspartate, as well as an efflux of L-serine, and is expressed in both astrocytes and neurons in mice (Hellsten et al., 2017), which was suggested by the authors to imply that SLC38A10 might be involved in neurotransmission. SLC38A10 has also been suggested to function as a transceptor (Tripathi et al., 2019) and be of importance for mitochondrial function and redox processes (Tripathi et al., 2021). The same authors found that SLC38A10 KO cells have an increased resistance to amino acid starvation, as well as an increased mTORC1 activation at basal level (Tripathi et al., 2022). *Slc38a10* mRNA expression was found in all mouse tissues investigated by Sundberg et al. (2008), with the highest expression in pituitary gland, eye and lung. *Slc38a10* expression is also found throughout the gastrointestinal tract in rats (Cedernaes et al., 2011), and found to have changed mRNA expression in mouse primary cortex cell cultures after amino acid starvation (Hellsten et al., 2018). *SLC38A10* has also been found to be associated with Alzheimer’s disease (Hashimoto et al., 2019), and a single-nucleotide polymorphism (SNP) and methylation differences in *SLC38A10* have been associated with frontotemporal dementia and schizophrenia (Ferrari et al., 2015; Montano et al., 2016).

SLC38A10 knockout mice have earlier been reported to have *weak and brittle bones* (Bassett et al., 2012) and a lower body weight than WT mice (Dickinson et al., 2016; Lindberg et al., 2022; Slc38a10 - solute carrier family 38, member 10, n.d). Moreover, amino acid transporters are known to affect brain function (Smith et al., 2001; Tărlungeanu et al., 2016; Hu et al., 2020), and behavioral studies are an important tool in studying such processes (Niv, 2021). In previous studies, SLC38A10 deficient mice have been characterized using several different behavioral test batteries (Dickinson et al., 2016; Lindberg et al., 2022; Slc38a10 - solute carrier family 38, member 10, n.d). Mixed results have been reported for the open field test, with studies showing no behavioral differences in SLC38A10 deficient mice (Dickinson et al., 2016; Slc38a10 - solute carrier family 38, member 10, n.d), but also results indicating a lower thigmotaxis behavior (Lindberg et al., 2022). Many of the previously used behavioral tests are designed to test predetermined mental states, such as *anxiety-like* behavior in the elevated plus maze (Walf and Frye, 2009; Hånell and Marklund, 2014). When using behavioral test batteries there is a risk of introducing multiple test effects (McIlwain et al., 2001).

Meyerson et al. (2006) proposed the implementation of a multivariate, not predetermined test which they called the concentric square filed test, later known as the multivariate concentric square field^TM^ (MCSF) test. In the MCSF, the animal is introduced to an arena with different zones associated to risk assessment, risk taking, exploration and shelter seeking, allowing the animal to freely choose where to go, resulting in a behavioral profile based on how the animals have moved in the arena (Bikovski et al., 2020). The MCSF has in previous studies been useful in studies of effects of mice domestication (e.g., Augustsson and Meyerson, 2004), for evaluation of specific gene functions (e.g., Wallén-Mackenzie et al., 2009) and for the characterization of specific disease models (e.g., Ekmark-Lewén et al., 2010; Stringer et al., 2017; Ekmark-Lewén et al., 2018). A complete behavioral profile of SLC38A10 mice has so far not been established and the aim of the present study was to behaviorally characterize mice deficient in SLC38A10 protein using the MCSF test.

## 2 Materials and methods

### 2.1 Animals

All experiments in the study were approved by the Uppsala Animal Ethical Committee (5.8.18-17636/2020) and followed Swedish Legislation on Animal Welfare. The animals were kept in an animal facility with a controlled environment, including humidity (45–65%), temperature (20–24°C), and ventilation, and with a 12-h light/dark cycle with lights on at 7 a.m. Animals were group housed in open cages, with two-five animals per Green Line cage (501–530 cm^2^), separated by sex, with wood-chip bedding, a cardboard house and paper as enrichment. Breeding was carried out in type III cages (800–820 cm^2^). Mice had daily supervision and *ad libitum* access to food (pellets R3, Lantmännen, Sweden) and water.

Heterozygous mice (B6Dnk; B6N-Slc38a10^tm2a(EUCOMM)^ ^Wtsi^/H) and control mice were bought from the International Mouse Phenotyping Consortium (IMPC)^1^ and kept for in house breeding. The first heterozygous males from that breeding were in turn bred with C57BL/6J females (Taconic M&B, Denmark). Further breeding was carried out from in house bred offspring. Genotyping was made as previously described (Lindberg et al., 2022).

In this paper, SLC38A10^–/–^ mice will be abbreviated as KO and SLC38A10^+/+^ as WT. Animals used (14 WT and 13 KO females; 14 WT and 11 KO males) came from heterozygous breeding and were 15–19 weeks of age.

### 2.2 Handling

Mice were trained to be handled and become accustomed to transportation to the experimental room in a bucket, in order to decrease the stressful response that these situations alone could cause. The handling started approximately 4.5 weeks before the MCSF test (at 10–14 weeks of age), with six training occasions during the first 2 weeks where one hand of the experimenter was placed in the cage for 5 min. During the third week of handling, each training occasion started with the experimenter’s hand in the cage for 2 min, and then the mouse was getting familiar to be placed on the arm of the experimenter and placed in the bucket, each of these occasions for 30 s each, for six consecutive days. Fourth week of training started with 2 min with the experimenter’s hand in the cage, 30 s per mouse in the bucket, and another 30 s walking around with each mouse in the bucket. The four remaining training occasions the last week were started with the experimenter’s hand in the cage for 1 min, and then walking around with each mouse in the bucket, increasing the duration and walking distance each day. After the last handling day, the mice were left undisturbed for 3 days before the start of the MCSF test. The bucket was cleaned with 10% ethanol between each cage that was trained/handled, and a separate bucket was used for males and females. Handling training was performed by the same experimenter that was running the MCSF test (FL).

### 2.3 The multivariate concentric square field^TM^ test

**FIGURE 1:**
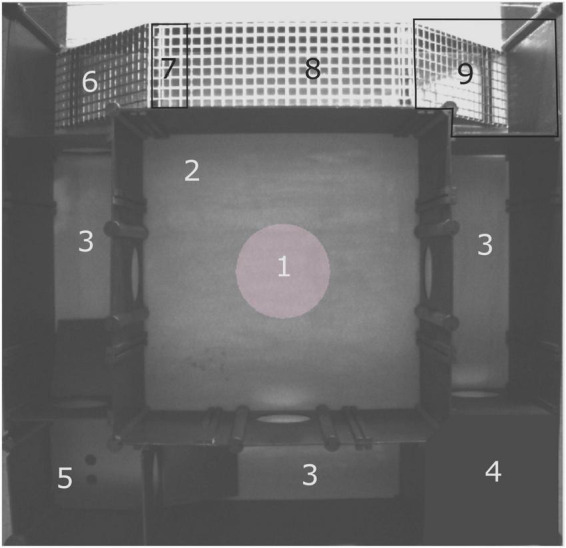
Layout of the multivariate concentric square field^TM^ (MCSF) test arena. (1) Central circle (CTRCI), (2) center, (3) corridors, (4) dark corner room (DCR), (5) hurdle with hole board and ramps to the adjacent corridors, (6) slope, (7) bridge entrance, (8) bridge and (9) beyond bridge zone.

The multivariate concentric square field^TM^ test is described in detail by Meyerson et al. (2006) and is used to characterize behavioral profiles from a single test trial (Bikovski et al., 2020). Briefly, the arena is 70 × 70 cm (Figure 1), and divided into zones that provide the animal with different explorative options, including sheltered, open and elevated areas. There is also a difference in illumination between areas, providing additional risk- or sheltered effects. The center of the arena is 40 × 40 cm, with a central circle of 16 cm in diameter in the middle. From the center, there are entries to the three corridors which leads to one sheltered corner, the dark corner room (DCR), the hurdle zone with a hole board for nose pokes, or a slope leading to the elevated and illuminated bridge. The slope beyond the bridge, here called *the beyond bridge zone*, distinguished this area from the risk-associated bridge. The hurdle zone had one ramp on each side to facilitate entries and exits. The illumination of the arena was as follows: 25 lux in the central circle, <1 lux in the DCR, 90–100 lux in the slope, 230–250 lux at the bridge entrance and 550 lux at the bridge. At the start of the test, the animal was placed in the arena facing the wall in the center without an entry to one of the corridors. Each trial was 20 min, and the arena was cleaned with 10% ethanol between each animal. Before the first trial of each day, an out-of-test mouse of the same sex as the ones to be tested explored the arena to avoid first-in-line effects. Testing occurred when the animals were 15–19 weeks of age (mean age 119.7 ± 7.3 days old), males were tested before females, and all tests were run during the light part of the light/dark cycle. Testing of the two genotypes was spread throughout the day. Due to technical difficulties, four mice were excluded from the analysis (2 WT males, 1 KO male, and 1 WT female), and gave rise to a final number of 13 WT and 13 KO females, and 12 WT and 10 KO males.

Trials were monitored, and rearing, nose-pokes, grooming and climbing were manually scored in an adjacent room to the test room using the Ethovision system (version 15, Noldus Information Technology, Wageningen, Netherlands). Ethovision version 16 was used for both manual scoring of zones and automatic tracking of total distance (cm) and mean velocity (cm/s) in the center and central circle. Occurrence and frequency (F) of zone visits, total duration (D, s) in each zone and latency (L, s) to first visit were scored for each zone. Urine and fecal boli were counted after each trial. Total activity (Tot Act, i.e., sum of all frequencies), percentage duration and frequency, duration per visit (D/F, s), risk/shelter index for frequencies and duration [(*F or D* bridge – *F or D* DCR)/(*F or D* bridge + *F or D* DCR)] and impulsive-like behavior [(latency to first (s) slope – latency to first (s) bridge)/latency to first (s) slope] were manually calculated (Roman et al., 2012).

### 2.4 Statistical analysis

Statistica 14 (TIBCO Software Inc., Tulsa, OK, United States) was used for statistical analyses unless otherwise stated, and a *p*-value of < 0.05 was considered statistically significant. A majority of the behavioral data was not normally distributed (Shapiro–Wilk normality test, *p* > 0.05), hence non-parametrical statistics were used. Parameters were analyzed with Mann–Whitney U-test comparing KO and WT with sex collapsed, KO and WT within the respective sex as well as males and females within genotype.

Occurrences were analyzed with Fisher’s exact test. Body weight data was normally distributed, and an unpaired t-test was therefore used. These analyses were made in in GraphPad Prism, version 5.02 (GraphPad software Inc., San Diego, CA, USA).

Longitudinal data sets (total activity and rearing over time) were analyzed for main effects and interactions in R 4.0.4 (R Core Team, 2021) with the nparLD package (Noguchi et al., 2012), with genotype and sex as between-subject factors and time as within-subject factor. Significant effects and interactions were further examined with *post hoc* Wilcoxon’s matched pairs test (within subject-dependent).

Furthermore, a trend analysis was performed (modified from Ekmark-Lewén et al., 2010; Meyerson et al., 2013) in order to detect differences in parameters belonging to the same functional category. 3–6 parameters per functional category (general activity (Tot Act, F TotCorr, D/F TotCorr [reversed], F center); exploratory activity [D TotCorr (reversed), D center (reversed), D hurdle, rearing, nose-pokes]; risk assessment (F slope, D slope, D/F slope, F bridge entrance, D bridge entrance, D/F bridge entrance); risk taking (F bridge, D bridge, D/F bridge, F central circle, D central circle, D/F central circle) and shelter seeking (F DCR, D DCR, D/F DCR) were used and ranked according to animal ID, summed up to sum rank scores and statistical analyses were made on the sum ranks for each functional category. Sum rank values were analyzed with Mann–Whitney U-test comparing KO and WT with sex collapsed, KO and WT within the respective sex as well as males and females within genotype.

Finally, a principal component analysis (PCA) was performed in SIMCA 17 (Sartorius Stedim Data Analytics AB, Umeå, Sweden) as a multivariate complement to the statistical analyses. Autofit was used to generate the model, and the model was based on frequency, duration and duration/visit in the zones with the corridors summed into total corridors as well as distance moved in the center, velocity in the central circle, and total activity. The PCA generates a two-dimensional score plot showing the individual animals and a corresponding loading plot showing the MCSF parameters.

## 3 Results

In agreement with previous reports (Dickinson et al., 2016; Lindberg et al., 2022; Slc38a10 - solute carrier family 38, member 10, n.d), SLC38A10 KO mice had a lower body weight than WT mice, which was true for both males and females (Supplementary Table 1).

### 3.1 No genotype effect on activity over time in the MCSF arena

**FIGURE 2:**
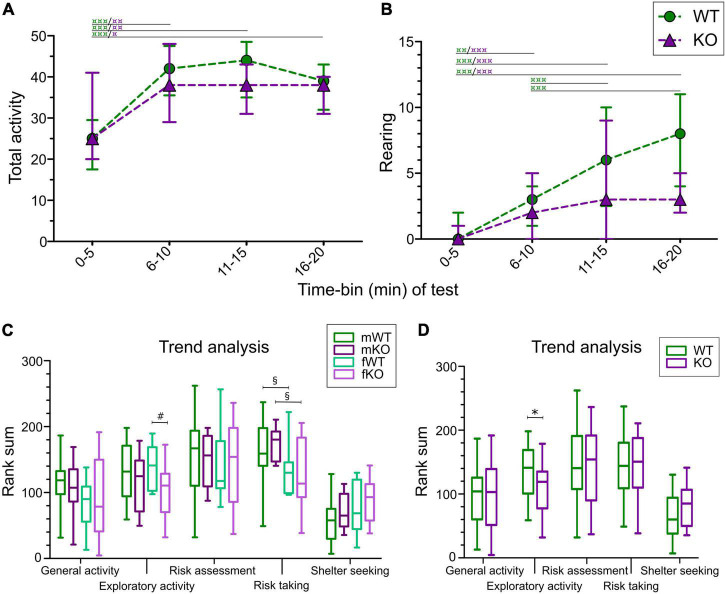
Activity and trend analysis of MCSF data in SLC38A10 KO and WT mice. **(A)** Total activity (Tot Act; sum of all frequencies) and **(B)** number of rearings of WT and KO mice divided into 5-min time-bins. Analysis was made with the nparLD-package in R, with *post hoc* Wilcoxon matched pairs test. 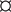
*p* < 0.05, 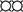
*p* < 0.01, and 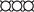
*p* < 0.001 within genotype over time. Data are presented as median with interquartile range. **(C,D)** Trend analysis of the functional categories general activity, exploratory activity, risk assessment, risk taking and shelter seeking analyzed **(C)** for each sex separately and **(D)** with sex collapsed. ^#^*p* < 0.05 comparing KO and WT mice within sex; §*p* < 0.05 denoting differences across sex and *p < 0.05 between KO and WT mice with sex collapsed. Data are presented as median with upper and lower quartiles and min and max values.

The activity of mice was studied by analyzing rearing as well as the sum of frequencies of visited zones (total activity) in each 5-min time-bin, resulting in four time-periods of 5 min each. As seen in Figure 2A, no genotype effect was seen in total activity; however, both WT and KO mice increased their activity over time compared to the first 5-min time-period (main effect of genotype *p* > 0.05, main effect of time *p* < 0.0001). Rearing also increased over time (Figure 2B; main effect of time *p* < 0.0001), while genotype had tendencies toward significance (main effect of genotype *p* = 0.0517). Descriptive statistics for each sex are summarized in Supplementary Table 2.

### 3.2 Genotype effects on the behavior in the MCSF

The descriptive data for male and female mice as well as with sex collapsed are shown in Supplementary Table 1. In females, the most pronounced genotype effect was seen in explorative parameters associated to the hurdle zone, where KO mice had lower duration, proportional duration (%D), duration per visit (D/F) and nose-pokes in the hole-board than WT mice. A trend could be seen for visits to the hurdle, with fewer visits in KO than WT females (*p* = 0.0568).

In males (Supplementary Table 1), visits to the slope and proportional visits to the slope (%F) were lower in KO mice, while the proportion of visits to the DCR was increased in KO males compared to WT. KO males also had an increased latency to the first visit of the beyond bridge zone. The calculated risk/shelter index for frequency was statistically different between male KO and WT mice, indicating fewer visits to the bridge than to the DCR in KO compared with WT males. Duration per visit to the slope, visits to the beyond bridge zone and velocity in the central circle were all close to the significance level (*p* = 0.0591, 0.0503, and 0.0610, respectively) with trends toward a longer duration per visit to the slope, fewer visits to the beyond bridge zone and a lower velocity in the central circle in KO mice.

In the analysis with sex collapsed (Supplementary Table 1), visits to the slope and proportional visits to the slope (%F) were decreased in KO mice. Trends could be seen for duration in hurdle (*p* = 0.0544) and for proportional duration in hurdle (D%, *p* = 0.0544), where KO mice had lower values for these parameters. The proportional visits (%F) to the DCR tended to be increased in KO mice but was just above the significance level (*p* = 0.0571). Rearing and nose-pokes in the sex collapsed comparison were close to the statistical significance level, where KO mice had a trend to fewer rearings and nose-pokes (*p* = 0.0659 and 0.0691, respectively). Another trend was the proportion of visits to the center, which tended to be increased in KO mice (*p* = 0.0571) compared to WT mice.

In the trend analysis, parameters are grouped into the functional categories: general activity, exploration, risk assessment, risk taking and shelter seeking, and compared between genotypes and sex. Exploration was lower in KO females compared to WT females (Figure 2C), and the same effect was seen with sex collapsed (Figure 2D), but not in the genotype comparison in only males. No genotype effect was seen for general activity, risk assessment, risk taking or shelter seeking in the trend analysis (Figures 2C,D).

**FIGURE 3:**
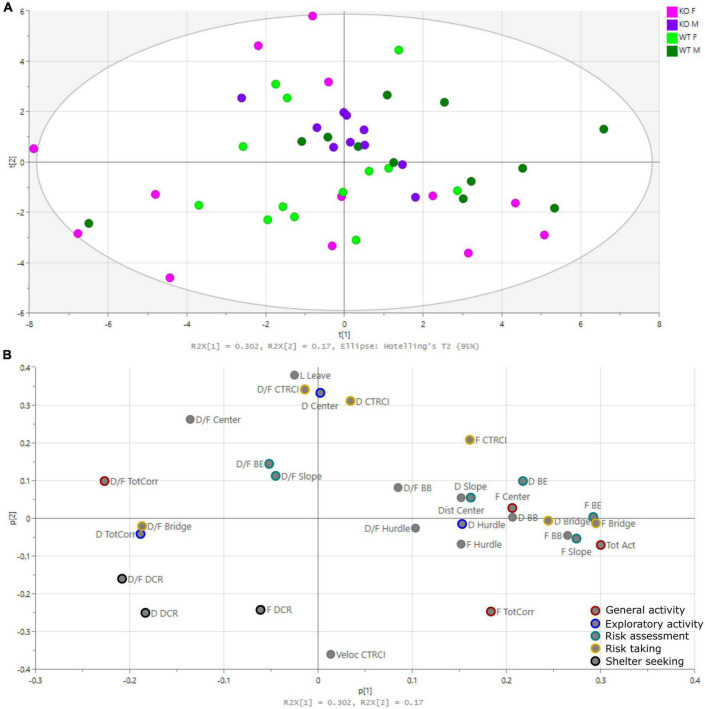
Principal component analysis (PCA). **(A)** Score plot showing the individual animals and **(B)** loading plot showing the parameters of importance for the individual loadings [one significant component with one non-significant component added: R2X(cum) = 0.472, Q2X(cum) = 0.278]. Parameters used in trend analysis are color-coded. BB, beyond bridge; BE, bridge entrance; CTRCI, central circle; DCR, dark corner room; Dist, distance; D, duration; D/F, duration/visit; F, frequency; L, latency; Tot Act, total activity; TotCorr, total corridors.

The PCA is shown in Figure 3 [one significant component with one non-significant component added; R2X(cum) = 0.472, Q2X(cum) = 0.278]. The score plot (Figure 3A), showing individual animals, did not clearly group genotypes together, but rather separated males and females. Male KO mice grouped close to the origo, while WT were more spread. Females on the other hand were more scattered from the origo, with KO females in all quadrants except the upper right one and WT females spread across all four quadrants. The loading plot (Figure 3B), showing parameters of importance for the individual loadings, showed that the latency in leaving the center (Lat leave), total activity (Tot Act), and parameters related to the DCR, bridge and central circle (CTRCI) contributed to the separation between males and females.

### 3.3 Sex differences

The male and female mice differed in behavior for some of the parameters. For instance, the genotype effect seen in female mice in hurdle-related parameters did not differ in males, where KO females had a lower duration and proportional duration (%D) compared with male KO mice. Moreover, nose-pokes in the hurdle zone differed between male and female mice, although only in the WT groups, with more nose-pokes in females than in males (Supplementary Table 1).

For distance moved in the center, WT females traveled a shorter distance than male mice. Latency to the first visit to the bridge entrance, proportion of visits to the slope (%F), and latencies to the first visits to the bridge and the beyond bridge zone were all increased in females compared to males, although not for both genotypes. A trend toward fewer visits to the bridge entrance, bridge and beyond bridge was seen in WT females compared to males (*p* = 0.0523, 0.0523, and 0.0597, respectively). There was also a trend toward a shorter duration and proportional duration (%D) in the center in KO females than males (*p* = 0.0575). Risk/shelter index for duration on bridge/DCR was trending to be lower in females than males (*p* = 0.0597, Supplementary Table 1).

A majority of the parameters regarding the central circle differed between male and female KO mice, with an increased latency to the first visit in females, as well as decreased duration, proportional duration (%D) and duration per visit in females. Moreover, velocity within the central circle was higher in females than in males (Supplementary Table 1). In the trend analysis, risk taking behavior was lower in females than in males, both in WT and KO mice (Figure 2C).

## 4 Discussion

In the current study, male and female SLC38A10 KO mice were characterized using the multivariate concentric square field^TM^ (MCSF) test. The outline of the MCSF arena, with zones associated with explorative incentives, risk and shelter, generates a behavioral profile in a single test session (Meyerson et al., 2006; Bikovski et al., 2020). Herein subtle behavioral differences between SLC38A10 KO and WT mice were revealed, but no distinct profile characterizing SLC38A10 deficient mice were found.

The trend analysis revealed a lower explorative behavior in KO than WT mice (Figure 2D), but there was only a trend toward lower number of rearings and nose-pokes in the hurdle hole board in KO relative to WT mice. While no effects on risk assessment, risk taking or shelter seeking were revealed in the trend analysis, there were tendencies toward lower risk assessment and increased shelter seeking in KO than WT mice based on specific parameters associated with risk assessment and shelter seeking, respectively (Supplementary Table 1). In our previous study of the same KO mouse model, the SLC38A10 deficient mice were found to have an increased explorative/risk-taking behavior in the open field test (Lindberg et al., 2022). The difference in results may be explained by the multivariate nature of the MCSF arena and the lack of possibilities to immediately oversee the arena compared to the open field test. An additional explanation might be the fact that the mice were older in the present study relative to previous studies (Dickinson et al., 2016; Lindberg et al., 2022; Slc38a10 - solute carrier family 38, member 10, n.d). Age is known to affect MCSF explorative strategies in rats (Lundberg et al., 2019) but has so far not been systematically investigated in mice. Nonetheless, these results together might suggest that SLC38A10 is involved in processes important for exploration and risk assessment, but that the effects seen are dependent on the environment. Adolescents over a range of species (including human) are known to be more risk taking than adults (reviewed by Spear, 2000); hence could a difference in this behavior between WT and KO mice possibly be greater in younger mice and thereby not be as evident in this study. Although it is hard to speculate about a possible effect of age, it is likely a parameter that makes the comparison between the previous open field study (Lindberg et al., 2022) and the current one even more difficult. The MCSF is designed to evaluate risk assessment, which tests used in previous studies (Dickinson et al., 2016; Lindberg et al., 2022; Slc38a10 - solute carrier family 38, member 10, n.d) are not, hence the data generated herein add an additional dimension to the behavioral profile of SLC38A10 deficient mice.

The beyond bridge zone (#9 in Figure 1) was not included in any functional category since it is scored for the first time (Wallén-Mackenzie et al., 2009; Ekmark-Lewén et al., 2010; Ekmark-Lewén et al., 2018; Bikovski et al., 2020). One could interpret the increased latency to the first visit to the beyond bridge zone in KO males as a sign of the lower explorative behavior seen for other parameters (Figures 2C,D). Since animals move from a risk area (bridge) to an unfamiliar area (beyond bridge), risk assessment is probably also involved, but due to the more sheltered part of the area, it is difficult to interpret visits (that tended to be lower in KO than WT males) and duration since we do not know what is motivating the animal to return to this zone. In the PCA loading plot (Figure 3), the beyond bridge zone parameters (duration and frequency) loaded close to parameters categorized as risk assessment and risk taking behaviors, suggesting that the beyond bridge zone could belong to the same category. This would be supported by the lower explorative strategies.

The lower explorative behavior in KO than WT mice revealed by the trend analysis (Figure 2D) was driven by the effect in female mice, as no difference between male mice was found. Exploration is generally higher in females than in males (Archer, 1975) and the same has been found for rats in the MCSF (Lundberg et al., 2018). In support of this finding, the explorative parameter nose-pokes in the hole board in the hurdle zone was higher in WT females than WT males (Supplementary Table 1), but other explorative parameters, e.g., rearing and latency to fully explore the arena were not affected by sex. Exploratory behavior in the MCSF has been reported to be affected by the estrous cycle in rats (Lundberg et al., 2017), but no reports of similar effects in female mice in the MCSF have been published to this date. Since estrous cycle was not controlled for, we cannot rule out that the estrous stage of females may have made an impact on their behavior and thereby might have caused a skewed balance in the experimental groups. A similar pattern could though be seen for male mice (Figure 2C), although not statistically significant, which disputes that the effect seen is only due to the estrous cycle itself. Amino acid metabolism is also known to be affected by sex hormones (Darst et al., 2019), which makes it possible that a SLC38A10 deficiency might affect females differently than males, depending on the involvement of this protein in amino acid sensing, transport and metabolism.

Parameters regarding risk assessment and shelter seeking were affected in males but not females, indicating that male KO mice were more cautious than their WT littermates. No previous studies characterizing SLC38A10 deficient mice have reported sex differences (Dickinson et al., 2016; Lindberg et al., 2022; Slc38a10 - solute carrier family 38, member 10, n.d) and, in agreement with the findings herein, general activity and rearings in the open field and general activity in the elevated plus maze and Y-maze did not differ between male and female mice (Lindberg et al., 2022). However, WT males were more risk taking than WT females and KO males were more risk taking than KO females, suggesting a more risk taking behavior in males, but which was stable across genotypes.

The differences in explorative behavior between KO and WT mice were not explained by differences in general activity. In both KO and WT mice, total activity and rearings increased over time (Figures 2A,B), suggesting that mice were a bit hesitant to explore the arena at first. In previous studies, total activity over time in mice has revealed mixed patterns. Most studies have reported that activity decreased over time (Wallén-Mackenzie et al., 2009; Ekmark-Lewén et al., 2010; Ekmark-Lewén et al., 2018) while one previous study showed results similar as in the present study (Wallén-Mackenzie et al., 2009). Moreover, the pattern found herein was similar to that found in the same KO mouse when exploring the central circle of the open field (Lindberg et al., 2022). Genotype effects for rearing over time was just above significance level, as well as for the total amount of rearing during the whole trial, indicating that vertical explorative behavior might be slightly decreased in SLC38A10 deficient mice. This finding contrasts a previous study using the same KO mouse but tested at a younger age, in which rearing in the open field test was unaffected by genotype, and generally higher during the first 5 min (Lindberg et al., 2022) than in the present study. This difference may again be explained by age as adolescent rats were found to rear more in the MCSF than adult rats (Lundberg et al., 2019), and similar results have been reported for mice as well (although between 8 and 11 months old mice, and not the primary outcome) (Ekmark-Lewén et al., 2018).

Relative to results from previous studies characterizing SLC38A10 deficient mice (Dickinson et al., 2016; Lindberg et al., 2022; Slc38a10 - solute carrier family 38, member 10, n.d), the results from the MCSF test give a more nuanced behavioral profile. Previous *in vitro* studies have reported an increased resistance to different types of stressors in KO cells (Tripathi et al., 2022, 2021), and based on the lack of a clear phenotype from the present study, challenging the KO model, e.g., with a stressor, could possibly distinguish KO mice from WT and give rise to a more pronounced behavioral profile in SLC38A10 deficient mice than the basal profile revealed herein.

## 5 Conclusion

Although a decrease in explorative behavior was found in KO mice in the trend analysis, suggesting that SLC38A10 might be of importance for exploration, an evident behavioral profile in SLC30A10 deficient mice relative to WT could not be found in a principal component analysis.

## Data availability statement

The original contributions presented in this study are included in the article/Supplementary material, further inquiries can be directed to the corresponding author.

## Ethics statement

The animal study was reviewed and approved by Uppsala Animal Ethical Committee.

## Author contributions

FL planned, performed, analyzed, and wrote the first draft of the manuscript. ER planned, analyzed, and wrote parts of the manuscript. RF planned and funded the study. All authors approved the final version of the manuscript.

## Footnotes

1
https://www.mousephenotype.org/data/genes/MGI:1919305
