# Supplementary material for: Behavioral profiling of SLC38A10 knockout mice using the multivariate concentric square fieldTM test
Source: Front Behav Neurosci. 2022 Dec 22;16:987037. doi: 10.3389/fnbeh.2022.987037 (PMC9815452; doi:10.3389/fnbeh.2022.987037)
Supplement: Supplementary file 1 [file Data_Sheet_1.zip › Supplementary Table 1-2.docx]

***Supplementary Material***

| **Table S1.** Descriptive statistics of each parameter in the multivariate concentric square field™ test, with sex separated and collapsed (n=12 WT and 10 KO males, 13 WT and 13 KO females). | | | | | | | | | | | | | | | | | | | | | | | | |
| --- | --- | --- | --- | --- | --- | --- | --- | --- | --- | --- | --- | --- | --- | --- | --- | --- | --- | --- | --- | --- | --- | --- | --- | --- |
| **Functional category** | **Parameters** | | | **WT** | |  |  | |  | |  |  | | **KO** | |  |  | |  | |  | |  |  |
|  |  |  |  | Males | |  | Females | |  | | Sex collapsed | | | Males | |  | Females | |  | | Sex collapsed | | |  |
|  |  |  |  | Median | | IQR | Median | | IQR | | Median | IQR | | Median | | IQR | Median | | IQR | | Median | | IQR |  |
| General activity | Tot Act | | | 150.5 | | 24.5 | 137.0 | | 34.0 | | 143.0 | 30.0 | | 141.0 | | 8.0 | 136.0 | | 56.0 | | 139.0 | | 43.0 |  |
|  | F totcorr | | | 40.5 | | 6.0 | 40.0 | | 10.0 | | 40.0 | 7.0 | | 41.5 | | 5.0 | 41.0 | | 22.0 | | 41.0 | | 15.0 |  |
|  | %F totcorr | | | 27.5 | | 6.2 | 30.8 | | 4.5 | | 29.1 | 6.2 | | 29.3 | | 2.8 | 30.1 | | 7.0 | | 29.5 | | 4.6 |  |
|  | F center | | | 27.0 | | 11.0 | 24.0 | | 6.0 | | 25.0 | 8.0 | | 30.0 | | 6.0 | 26.0 | | 10.0 | | 27.0 | | 9.0 |  |
|  | %F center | | | 19.1 | | 5.2 | 16.8 | | 5.6 | | 17.6 | 6.2 | | 20.9 | | 4.9 | 19.2 | | 3.9 | | 20.7 | | 4.5 |  |
|  | D center | | | 232.3 | | 83.8 | 162.9 | | 76.7 | | 184.9 | 89.8 | | 247.7 | | 56.4 | 176.5 | | 72.9 | | 210.3 | | 108.4 |  |
|  | %D center | | | 19.4 | | 7.0 | 13.6 | | 6.4 | | 15.4 | 7.5 | | 20.6 | | 4.7 | 14.7 | | 6.1 | | 17.5 | | 9.0 |  |
|  | D/F center | | | 8.5 | | 4.3 | 7.5 | | 4.2 | | 8.2 | 4.5 | | 9.2 | | 4.0 | 6.0 | | 4.4 | | 8.4 | | 5.8 |  |
|  | Distance center | | | 1483.0 | | 449.1 | 1167.6 | | 218.1^§^ | | 1226.8 | 386.9 | | 1435.1 | | 313.6 | 1271.7 | | 499.0 | | 1323.3 | | 379.7 |  |
|  | Velocity center | | | 6.3 | | 1.7 | 6.8 | | 3.1 | | 6.3 | 2.3 | | 5.6 | | 1.2 | 7.7 | | 3.6 | | 5.8 | | 3.2 |  |
| Exploratory activity | L leave | | | 131.3 | | 125.2 | 53.8 | | 42.3 | | 73.2 | 114.9 | | 129.1 | | 48.1 | 33.2 | | 78.1 | | 90.6 | | 109.4 |  |
|  | D totcorr | | | 345.0 | | 116.9 | 423.3 | | 192.5 | | 368.7 | 120.5 | | 365.3 | | 108.2 | 402.1 | | 101.5 | | 380.7 | | 108.2 |  |
|  | %D totcorr | | | 28.7 | | 9.7 | 35.3 | | 16.0 | | 30.7 | 10.0 | | 30.4 | | 9.0 | 33.5 | | 8.5 | | 31.7 | | 9.0 |  |
|  | D/F totcorr | | | 8.2 | | 3.0 | 9.6 | | 3.5 | | 8.6 | 2.6 | | 8.9 | | 2.3 | 10.2 | | 6.5 | | 9.3 | | 5.1 |  |
|  | L hurdle | | | 261.3 | | 246.5 | 313.2 | | 177.4 | | 291.7 | 172.9 | | 246.6 | | 225.0 | 279.1 | | 590.0 | | 279.1 | | 305.9 |  |
|  | F hurdle | | | 9.0 | | 2.0 | 9.0 | | 4.0 | | 9.0 | 3.0 | | 8.0 | | 3.0 | 6.0 | | 6.0 | | 7.0 | | 6.0 |  |
|  | %F hurdle | | | 5.9 | | 2.3 | 6.3 | | 2.5 | | 5.9 | 2.1 | | 5.6 | | 2.5 | 5.6 | | 3.3 ^#^ | | 5.6 | | 2.0 |  |
|  | D hurdle | | | 106.4 | | 30.1 | 87.6 | | 41.0 | | 105.9 | 33.5 | | 104.5 | | 37.5 ^§^ | 51.7 | | 88.2 ^#^ | | 88.2 | | 91.1 |  |
|  | %D hurdle | | | 8.9 | | 2.5 | 7.3 | | 3.4 | | 8.8 | 2.8 | | 8.7 | | 3.1 ^§^ | 4.3 | | 7.4 ^#^ | | 7.4 | | 7.6 |  |
|  | D/F hurdle | | | 10.9 | | 2.9 | 10.8 | | 3.6 | | 10.8 | 3.2 | | 11.1 | | 8.1 | 7.9 | | 9.0 ^#^ | | 8.4 | | 10.3 |  |
|  | Occ hurdle | | | 12/12 | |  | 13/13 | |  | | 25/25 |  | | 10/10 | |  | 11/13 | |  | | 21/23 | |  |  |
|  | L explored arena | | | 550.3 | | 272.0 | 595.2 | | 234.3 | | 552.5 | 249.1 | | 585.0 | | 235.8 | 679.5 | | 336.3 | | 641.1 | | 307.3 |  |
|  | Occ. visit all zones | | | 11/12 | |  | 12/13 | |  | | 23/25 |  | | 10/10 | |  | 10/13 | |  | | 20/23 | |  |  |
|  | Rearing | | | 18.5 | | 16.5 | 19.0 | | 7.0 | | 19.0 | 9.0 | | 11.5 | | 19.0 | 8.0 | | 15.0 | | 10.0 | | 16.0 |  |
|  | Occ. rearing | | | 12/12 | |  | 13/13 | |  | | 25/25 |  | | 9/10 | |  | 13/13 | |  | | 22/23 | |  |  |
|  | Nose-pokes | | | 2.5 | | 5.5 | 6.0 | | 5.0 ^§^ | | 5.0 | 7.0 | | 3.5 | | 5.0 | 1.0 | | 3.0 ^#^ | | 2.0 | | 4.0 |  |
|  | Occ. Nose-pokes | | | 7/12 | |  | 11/13 | |  | | 18/25 |  | | 7/10 | |  | 7/13 | |  | | 14/23 | |  |  |
| Risk assessment | L slope | | | 194.4 | | 234.8 | 329.4 | | 156.5 | | 278.5 | 218.8 | | 222.1 | | 134.6 | 306.2 | | 339.1 | | 235.7 | | 297.9 |  |
|  | F slope | | | 15.5 | | 7.0 | 15.0 | | 1.0 | | 15.0 | 2.0 | | 12.5 | | 1.0 ^#^ | 14.0 | | 5.0 | | 13.0 | | 3.0 * |  |
|  | %F slope | | | 10.2 | | 1.7 | 10.1 | | 2.5 | | 10.1 | 1.7 | | 8.5 | | 1.1 ^###^ | 10.3 | | 3.0 ^§§^ | | 9.3 | | 2.0 * |  |
|  | D slope | | | 85.5 | | 62.5 | 68.8 | | 26.4 | | 76.9 | 50.5 | | 79.9 | | 28.3 | 77.6 | | 49.6 | | 77.6 | | 38.4 |  |
|  | %D slope | | | 7.1 | | 5.2 | 5.7 | | 2.2 | | 6.4 | 4.2 | | 6.7 | | 2.4 | 6.5 | | 4.1 | | 6.5 | | 3.2 |  |
|  | D/F slope | | | 5.2 | | 1.8 | 5.5 | | 2.2 | | 5.5 | 2.1 | | 6.2 | | 2.3 | 5.5 | | 2.6 | | 6.0 | | 2.1 |  |
|  | L bridge entrance | | | 222.6 | | 201.5 | 420.5 | | 132.8 ^§^ | | 343.8 | 229.5 | | 242.4 | | 75.9 | 338.1 | | 191.7 | | 258.2 | | 203.7 |  |
|  | F bridge entrance | | | 16.5 | | 8.0 | 14.0 | | 5.0 | | 14.0 | 6.0 | | 15.5 | | 3.0 | 16.0 | | 8.0 | | 16.0 | | 4.0 |  |
|  | %F bridge entrance | | | 11.1 | | 2.9 | 10.3 | | 2.6 | | 11.0 | 2.8 | | 11.3 | | 2.5 | 11.6 | | 3.3 | | 11.6 | | 3.2 |  |
|  | D bridge entrance | | | 49.3 | | 18.8 | 43.2 | | 20.9 | | 48.5 | 16.0 | | 50.5 | | 14.2 | 49.9 | | 33.7 | | 50.0 | | 22.8 |  |
|  | %D bridge entrance | | | 4.1 | | 1.6 | 3.6 | | 1.7 | | 4.0 | 1.3 | | 4.2 | | 1.2 | 4.2 | | 2.8 | | 4.2 | | 1.9 |  |
|  | D/F bridge entrance | | | 3.1 | | 1.2 | 3.5 | | 0.7 | | 3.3 | 0.9 | | 3.6 | | 0.9 | 3.1 | | 0.7 | | 3.2 | | 1.1 |  |
| Risk taking | L bridge | | | 225.0 | | 201.4 | 423.1 | | 133.0 ^§^ | | 345.7 | 229.2 | | 243.8 | | 76.6 | 339.5 | | 191.9 | | 261.0 | | 206.6 |  |
|  | F bridge | | | 15.5 | | 10.0 | 12.0 | | 5.0 | | 14.0 | 6.0 | | 13.5 | | 4.0 | 14.0 | | 9.0 | | 14.0 | | 6.0 |  |
|  | %F bridge | | | 9.9 | | 4.9 | 8.9 | | 2.6 | | 9.4 | 3.5 | | 9.0 | | 3.0 | 9.2 | | 2.1 | | 9.1 | | 2.5 |  |
|  | D bridge | | | 146.1 | | 85.8 | 127.7 | | 44.4 | | 135.1 | 48.5 | | 133.2 | | 58.1 | 107.3 | | 55.7 | | 124.7 | | 67.2 |  |
|  | %D bridge | | | 12.2 | | 7.1 | 10.6 | | 3.7 | | 11.3 | 4.0 | | 11.1 | | 4.8 | 8.9 | | 4.6 | | 10.4 | | 5.6 |  |
|  | D/F bridge | | | 9.8 | | 1.6 | 10.7 | | 1.4 | | 10.2 | 1.4 | | 11.2 | | 2.7 | 9.6 | | 5.0 | | 10.6 | | 4.2 |  |
|  | L CTRCI | | | 7.4 | | 93.0 | 27.5 | | 67.3 | | 17.7 | 89.5 | | 7.4 | | 29.7 | 167.3 | | 574.2 ^§§^ | | 31.3 | | 203.3 |  |
|  | F CTRCI | | | 8.0 | | 9.0 | 7.0 | | 3.0 | | 7.0 | 7.0 | | 11.0 | | 8.0 | 9.0 | | 9.0 | | 10.0 | | 10.0 |  |
|  | %F CTRCI | | | 5.5 | | 4.3 | 5.1 | | 4.5 | | 5.3 | 4.5 | | 7.7 | | 4.0 | 6.4 | | 4.6 | | 6.4 | | 5.8 |  |
|  | D CTRCI | | | 8.9 | | 14.6 | 7.2 | | 5.7 | | 7.5 | 7.4 | | 9.5 | | 16.2 | 6.2 | | 6.3 ^§^ | | 7.9 | | 9.5 |  |
|  | %D CTRCI | | | 0.7 | | 1.2 | 0.6 | | 0.5 | | 0.6 | 0.6 | | 0.8 | | 1.3 | 0.5 | | 0.5 ^§^ | | 0.7 | | 0.8 |  |
|  | D/F CTRCI | | | 1.0 | | 0.7 | 0.9 | | 0.9 | | 0.9 | 0.8 | | 1.5 | | 0.9 | 0.6 | | 0.4 ^§^ | | 0.9 | | 1.2 |  |
|  | Distance CTRCI | | | 124.9 | | 154.0 | 108.4 | | 73.8 | | 116.7 | 101.1 | | 137.4 | | 93.3 | 124.4 | | 114.3 | | 124.4 | | 112.7 |  |
|  | Velocity CTRCI | | | 14.5 | | 7.5 | 11.8 | | 11.3 | | 11.9 | 9.2 | | 8.9 | | 8.7 | 22.0 | | 15.5 ^§^ | | 14.3 | | 14.6 |  |
|  | Occ. CTRCI | | | 11/12 | |  | 12/13 | |  | | 23/25 |  | | 10/10 | |  | 13/13 | |  | | 23/23 | |  |  |
| Shelter seeking | L DCR | | | 338.5 | | 314.4 | 247.6 | | 336.6 | | 316.7 | 288.1 | | 399.1 | | 373.9 | 200.2 | | 261.3 | | 208.0 | | 352.5 |  |
|  | F DCR | | | 7.0 | | 3.5 | 7.0 | | 3.0 | | 7.0 | 3.0 | | 9.0 | | 2.0 | 9.0 | | 2.0 | | 9.0 | | 2.0 |  |
|  | %F DCR | | | 4.8 | | 2.4 | 5.7 | | 3.3 | | 4.9 | 2.1 | | 6.3 | | 0.4 ^#^ | 6.4 | | 3.0 | | 6.3 | | 1.5 |  |
|  | D DCR | | | 75.6 | | 55.1 | 96.3 | | 163.3 | | 78.6 | 67.3 | | 91.9 | | 69.7 | 135.7 | | 93.0 | | 131.0 | | 100.9 |  |
|  | %D DCR | | | 6.3 | | 4.6 | 8.0 | | 13.6 | | 6.5 | 5.6 | | 7.7 | | 5.8 | 11.3 | | 7.7 | | 10.9 | | 8.4 |  |
|  | D/F DCR | | | 11.0 | | 7.4 | 12.7 | | 12.8 | | 12.4 | 7.1 | | 11.4 | | 4.8 | 17.3 | | 12.5 | | 14.2 | | 9.8 |  |
| Other | L beyond bridge | | | 240.5 | | 183.0 | 523.4 | | 146.0 ^§§§^ | | 409.1 | 303.7 | | 507.6 | | 346.6 ^##^ | 497.7 | | 238.2 | | 507.6 | | 315.5 |  |
|  | F beyond bridge | | | 7.0 | | 5.5 | 4.0 | | 3.0 | | 5.0 | 4.0 | | 4.0 | | 2.0 | 4.0 | | 4.0 | | 4.0 | | 4.0 |  |
|  | %F beyond bridge | | | 4.2 | | 2.5 | 2.9 | | 2.0 | | 3.4 | 2.2 | | 2.9 | | 1.6 | 2.8 | | 2.0 | | 2.9 | | 1.9 |  |
|  | D beyond bridge | | | 108.5 | | 106.3 | 74.2 | | 54.7 | | 92.2 | 92.6 | | 94.6 | | 82.3 | 69.2 | | 104.0 | | 71.9 | | 114.4 |  |
|  | %D beyond bridge | | | 9.0 | | 8.9 | 6.2 | | 4.6 | | 7.7 | 7.7 | | 7.9 | | 6.9 | 5.8 | | 8.7 | | 6.0 | | 9.5 |  |
|  | D/F beyond bridge | | | 15.1 | | 7.9 | 17.7 | | 7.9 | | 17.1 | 8.2 | | 17.2 | | 8.1 | 9.0 | | 14.1 | | 16.4 | | 14.6 |  |
|  | Grooming | | | 2.0 | | 1.0 | 2.0 | | 5.0 | | 2.0 | 2.0 | | 2.0 | | 2.0 | 2.0 | | 3.0 | | 2.0 | | 2.0 |  |
|  | Occ. grooming | | | 11/12 | |  | 9/13 | |  | | 20/25 |  | | 10/10 | |  | 8/13 | |  | | 18/23 | |  |  |
|  | Climbing | | | 0.5 | | 1.0 | 0.0 | | 0.0 | | 0.0 | 1.0 | | 0.0 | | 1.0 | 0.0 | | 1.0 | | 0.0 | | 1.0 |  |
|  | Occ. climbing | | | 6/12 | |  | 2/13 | |  | | 8/25 |  | | 3/10 | |  | 4/13 | |  | | 7/23 | |  |  |
|  | Boli | | | 0.0 | | 3.0 | 0.0 | | 0.5 | | 0.0 | 0.5 | | 0.0 | | 1.0 | 0.0 | | 0.5 | | 0.0 | | 1.0 |  |
|  | Occ. boli | | | 3/12 | |  | 3/13 | |  | | 6/25 |  | | 3/10 | |  | 3/13 | |  | | 6/23 | |  |  |
|  | Urine | | | 0.0 | | 0.0 | 0.0 | | 0.0 | | 0.0 | 0.0 | | 0.0 | | 0.0 | 0.0 | | 0.0 | | 0.0 | | 0.0 |  |
|  | Occ. urine | | | 1/12 | |  | 0/13 | |  | | 1/25 |  | | 0/10 | |  | 0/13 | |  | | 0/23 | |  |  |
|  | Body weight (mean±SD) | | | 29.5 | | 1.6 | 23.5 | | 1.5 | |  |  | | 25.9 | | 2.4 ^###^ | 19.1 | | 0.9 ^####^ | |  | |  |  |
|  | F risk/shelter index | | | 0.3 | | 0.4 | 0.2 | | 0.3 | | 0.3 | 0.3 | | 0.2 | | 0.2 ^#^ | 0.1 | | 0.4 | | 0.2 | | 0.3 |  |
|  | D risk/shelter index | | | 0.4 | | 0.5 | 0.1 | | 0.5 | | 0.2 | 0.6 | | 0.2 | | 0.4 | -0.1 | | 0.6 | | 0.1 | | 0.5 |  |
|  | Slope/bridge interval | | | -0.1 | | 0.5 | -0.1 | | 0.6 | | -0.1 | 0.6 | | 0.0 | | 0.0 | -0.1 | | 0.4 | | 0.0 | | 0.3 |  |
| Behavioral parameters recorded during the 20-min trial of the MCSF test. Values represent median and interquartile range (IQR), except for body weight which are presented as means with standard deviation. Occurrence (Occ.) is shown for the zones that were not visited by all animals in each group, and for behaviors not performed by all animals in each group (Fisher's exact test). *p < 0.05, **p < 0.01, ***p < 0.001 comparing WT and KO mice with sex collapsed; #p < 0.05, ##p < 0.01, ###p < 0.001 comparing WT and KO mice within sex and §p < 0.05, §§p < 0.01, §§§p < 0.001 comparing across sex (Mann-Whitney U-test). Body weight was only analyzed for each sex separately (unpaired t-test). Abbreviations: CTRCI. central circle; DCR, dark corner room; D, duration (s); D/F, duration per visit (s); F, frequency; L, latency (s); Occ, occurrence; Tot Act, total activity i.e., the sum of all frequencies; TOTCORR, total corridor i.e. the sum of all corridors. | | | | | | | | | | | | | | | | | | | | | | | | |
| **Table S2.** Descriptive statistics of Tot Act and rearing over time for males and females (n=12 WT and 10 KO males, 13 WT and 13 KO females). | | | | | | | | | | | | | | | | | | | | | |  |  |  |
| **Parameter** | | **Time-point (min)** | **WT** | |  | | |  | |  | | | **KO** | |  | | |  | |  | |  |  |  |
|  |  |  | Males | |  | | | Females | |  | | | Males | |  | | | Females | |  | |  |  |  |
|  |  |  | Median | | IQR | | | Median | | IQR | | | Median | | IQR | | | Median | | IQR | |  |  |  |
| Tot Act | | 0-5 | 28.5 | | 14.5 | | | 24.0 | | 7.0 | | | 29.0 | | 16.0 | | | 24.0 | | 24.0 | |  |  |  |
|  |  | 6-10 | 45.0 | | 6.5 | | | 38.0 | | 10.0 | | | 40.0 | | 11.0 | | | 38.0 | | 21.0 | |  |  |  |
|  |  | 11-15 | 47.0 | | 10.0 | | | 41.0 | | 11.0 | | | 38.0 | | 5.0 | | | 36.0 | | 15.0 | |  |  |  |
|  |  | 16-20 | 39.5 | | 6.5 | | | 36.0 | | 15.0 | | | 38.5 | | 6.0 | | | 34.0 | | 21.0 | |  |  |  |
| Rearing | | 0-5 | 0.0 | | 1.5 | | | 0.0 | | 2.0 | | | 0.0 | | 1.0 | | | 1.0 | | 1.0 | |  |  |  |
|  |  | 6-10 | 2.5 | | 3.5 | | | 3.0 | | 3.0 | | | 3.5 | | 5.0 | | | 2.0 | | 4.0 | |  |  |  |
|  |  | 11-15 | 7.0 | | 7.5 | | | 6.0 | | 7.0 | | | 3.5 | | 9.0 | | | 3.0 | | 7.0 | |  |  |  |
|  |  | 16-20 | 8.0 | | 9.0 | | | 6.0 | | 5.0 | | | 3.5 | | 3.0 | | | 3.0 | | 3.0 | |  |  |  |
| Tot Act and rearing over time, divided into five-minute time-bins. Values represent median and interquartile range (IQR). Abbreviation: Tot Act, total activity, i.e., the sum of all frequencies. | | | | | | | | | | | | | | | | | | | | | |  |  |  |
